# MicroRNA-122 affects cell aggressiveness and apoptosis by targeting PKM2 in human hepatocellular carcinoma

**DOI:** 10.3892/or.2021.8073

**Published:** 2021-05-06

**Authors:** Qiuran Xu, Meiqi Zhang, Jianfeng Tu, Linxiao Pang, Wenwei Cai, Xin Liu

Oncol Rep 34: 2054-2064, 2015; DOI: 10.3892/or.2015.4175

Following the publication of the above article, an interested reader drew to the authors’ attention that Figs. 6C and 7A apparently contained overlapping panels, suggesting that these data for purportedly different experiments had been derived from the same original source. The wound-healing result for the anti-miR-122+siRNA PKM2 experiment in the lower panel of Fig. 6C and the transwell invasion result for the anti-miR-122+siRNA PKM2 experiment in Fig. 7A were erroneously selected. Accordingly, the authors repeated these assays and were able to confirm that the results were in accordance with the published results.

Consequently, the corrected versions of Figs. 6 and 7, containing the replacement data for Figs. 6C and 7A, are shown opposite. It should be emphasized that the inadvertent errors that occurred during the compilation of these figures did not affect the research results or the conclusions of this article. The authors all agree to this Corrigendum, and are grateful to the Editor of *Oncology Reports* for allowing them to have the opportunity to correct these errors. The authors also apologize to the readership for any inconvenience these errors may have caused.

## Figures and Tables

**Figure 6.**
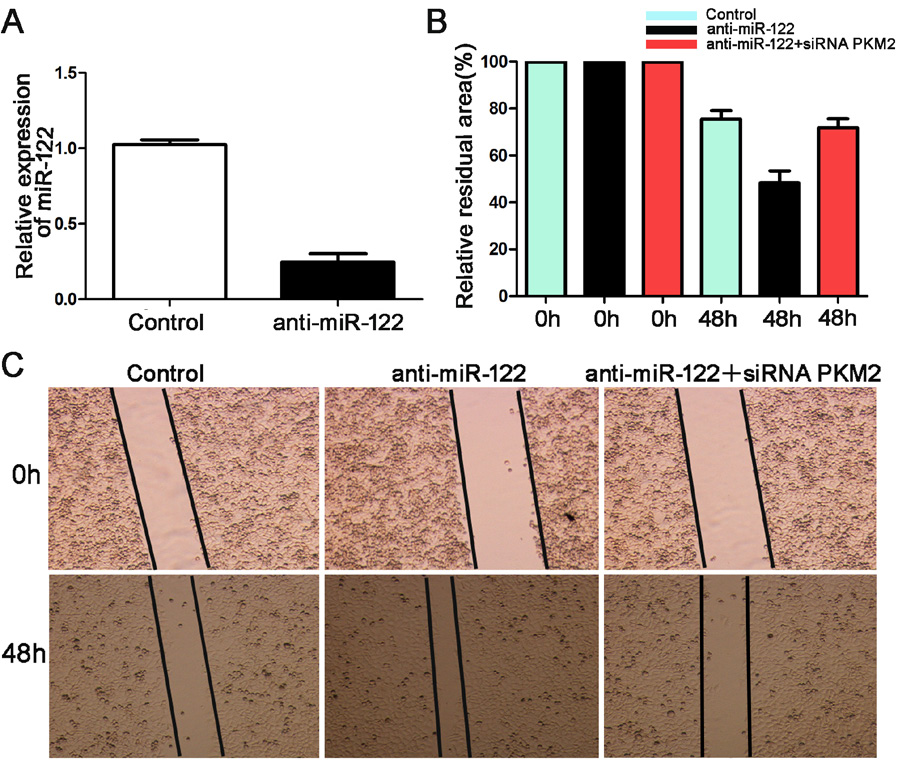
MiR-122 regulates the invasion and migration of Hep3B cells by targeting PKM2. (A) Hep3B cells transfected with the negative control and anti-miR-122 were subjected to qRT-PCR for miR-122 (n=6; *P<0.05). (B and C) Wound healing assays revealed that knockdown of miR-122 increased the migration of Hep3B cells, while, siRNA PKM2 was found to reduce the inhibition effect on migration of anti-miR-122 in the Hep3B cells, *P<0.05 by t-test.

**Figure 7.**
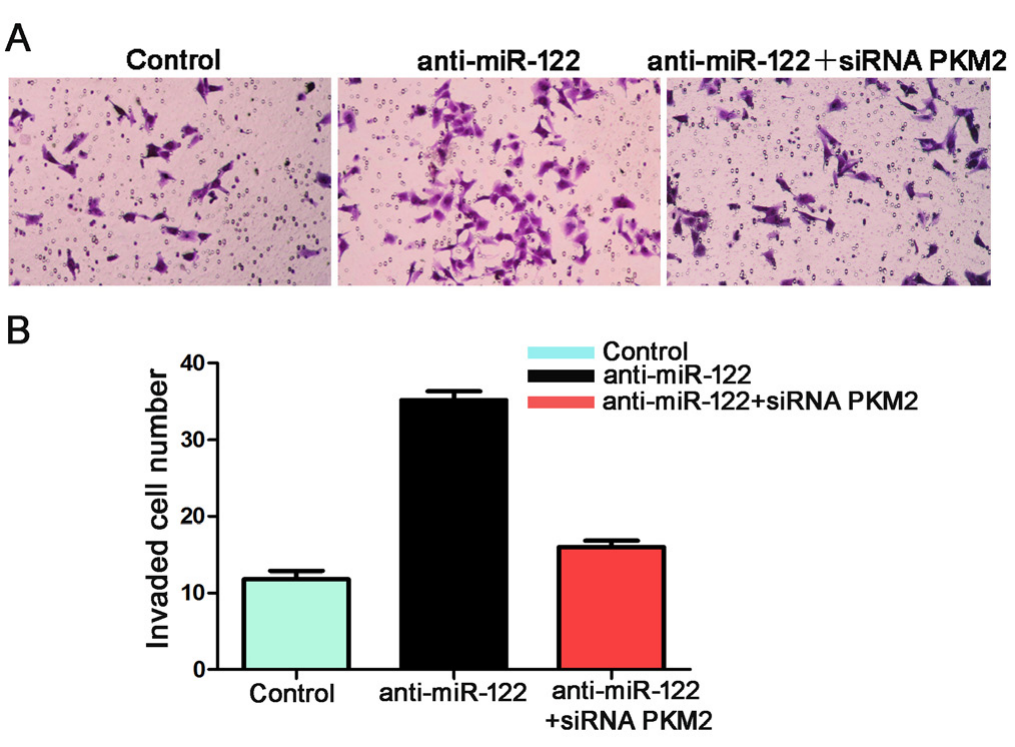
The number of invaded Hep3B cells in the anti-miR-122 group was significantly more than the number in the control group. n=6 repeats with similar results; *P<0.05 by t-test. Data are expressed as the mean ± SEM.

